# In Vitro Cellular Uptake and Transfection of Oligoarginine-Conjugated Glycol Chitosan/siRNA Nanoparticles

**DOI:** 10.3390/polym13234219

**Published:** 2021-12-01

**Authors:** Eun-Ju Jeong, Jangwook Lee, Hyun-Seung Kim, Kuen-Yong Lee

**Affiliations:** 1 Department of Bioengineering, Hanyang University, 222 Wangsimni-ro, Seongdong-gu, Seoul 04763, Korea; ejeong@supernovabio.com (E.-J.J.); jlee@kribb.re.kr (J.L.); hyuns9393@naver.com (H.-S.K.); 2 Supernova Bio, 67 Seobinggo-ro, Yongsan-gu, Seoul 04385, Korea; 3 Biotherapeutics Translational Research Center, Korea Research Institute of Bioscience and Biotechnology, 125 Gwahak-ro, Yuseong-gu, Daejeon 34141, Korea; 4 Institute of Nano Science and Technology, Hanyang University, 222 Wangsimni-ro, Seongdong-gu, Seoul 04763, Korea

**Keywords:** glycol chitosan, oligoarginine, siRNA, gene therapy

## Abstract

Chitosan and its derivatives have been extensively utilized in gene delivery applications because of their low toxicity and positively charged characteristics. However, their low solubility under physiological conditions often limits their application. Glycol chitosan (GC) is a derivative of chitosan that exhibits excellent solubility in physiological buffer solutions. However, it lacks the positive characteristics of a gene carrier. Thus, we hypothesized that the introduction of oligoarginine peptide to GC could improve the formation of complexes with siRNA, resulting in enhanced uptake by cells and increased transfection efficiency in vitro. A peptide with nine arginine residues and 10 glycine units (R_9_G_10_) was successfully conjugated to GC, which was confirmed by infrared spectroscopy, ^1^H NMR spectroscopy, and elemental analysis. The physicochemical characteristics of R_9_G_10_-GC/siRNA complexes were also investigated. The size and surface charge of the R_9_G_10_-GC/siRNA nanoparticles depended on the amount of R_9_G_10_ coupled to the GC. In addition, the R_9_G_10_-GC/siRNA nanoparticles showed improved uptake in HeLa cells and enhanced in vitro transfection efficiency while maintaining low cytotoxicity determined by the MTT assay. Oligoarginine-modified glycol chitosan may be useful as a potential gene carrier in many therapeutic applications.

## 1. Introduction

RNA interference (RNAi) is an innovative approach for selective and specific gene silencing and has demonstrated potential for the treatment of various diseases [1,2,3,4,5]. In this pathway, RNA-induced silencing complex (RISC), formed from double-stranded small interfering RNA (siRNA), degrades target mRNA and inhibits protein synthesis [6,7]. RNAi has often been used to treat many disorders, including cancer, infectious diseases, and rare genetic disorders [8,9,10,11]. However, low stability of naked siRNA in the blood flow, intracellular absorption, and rapid degradation are still challenging to achieve successful therapeutic RNAi. Thus, it is critical to utilize a proper delivery system to enhance the stability and intracellular uptake of siRNA [12,13]. Many siRNA-based nanotherapeutics have been investigated clinically to treat patients and several of them are approved by FDA (e.g., ONPATTRO^®^, Alnylam, Cambridge, MA, USA) [14,15,16].

Although viral systems have high transfection efficiency, many safety issues, including unexpected inflammation, undesirable immune responses, and carcinogenesis, have limited their applications [17]. Non-viral systems including cationic polymers with low immune responses can be easily synthesized and modified [18,19]. Cationic polymers have been widely used for siRNA delivery, as they can easily form complexes with siRNA via electrostatic interactions and are rapidly taken up by cells [20,21]. However, the repeated administration of cationic polymers could result in unwanted toxicity and the release of siRNA from the complexes could be slow due to the permanent cationic charges [22]. Thus, self-catalyzed, degradable polymer systems were designed to mimic the escape mechanism of the influenza virus from the endosome and to regulate the timed release of siRNA in the cytosol [23]. Organic/inorganic hybrid systems were also developed to facilitate endosomal escape of siRNA [24].

Chitosan, the second most abundant natural polymer, can be obtained from chitin [25,26]. Chitosan is a widely used gene carrier, as it also demonstrates positively charged characteristics that enable interaction with negatively charged nucleic acids via electrostatic interactions [27,28,29]. Chitosan exhibits the potential for effective siRNA silencing in vitro and in vivo [30,31,32]. However, chitosan is typically soluble under acidic conditions, which may limit its medical applications in the body. Glycol chitosan (GC) is a derivative chitosan that is freely soluble in distilled water as well as in physiological buffer solution. Although GC has been also used for siRNA delivery, it inherently lacks positively charged characteristics compared with chitosan [33].

Various arginine-based systems have been designed and fabricated for gene delivery, inspired by excellent trans-membrane efficiency of cell-penetrating peptides (CPPs) [34]. Oligoarginine has often been used to develop non-viral gene delivery methods [34,35]. Octaarginine (R_8_) and nonaarginine (R_9_) were useful as potent CPPs to improve the intracellular delivery of drugs and genes [36,37,38,39]. Octaarginine-modified chitosan enhanced in vitro transfection efficiency of DNA [36] and nonaarginine-modified chitosan showed excellent gene silencing effect while maintaining low cytotoxicity [39].

**Figure 1:**
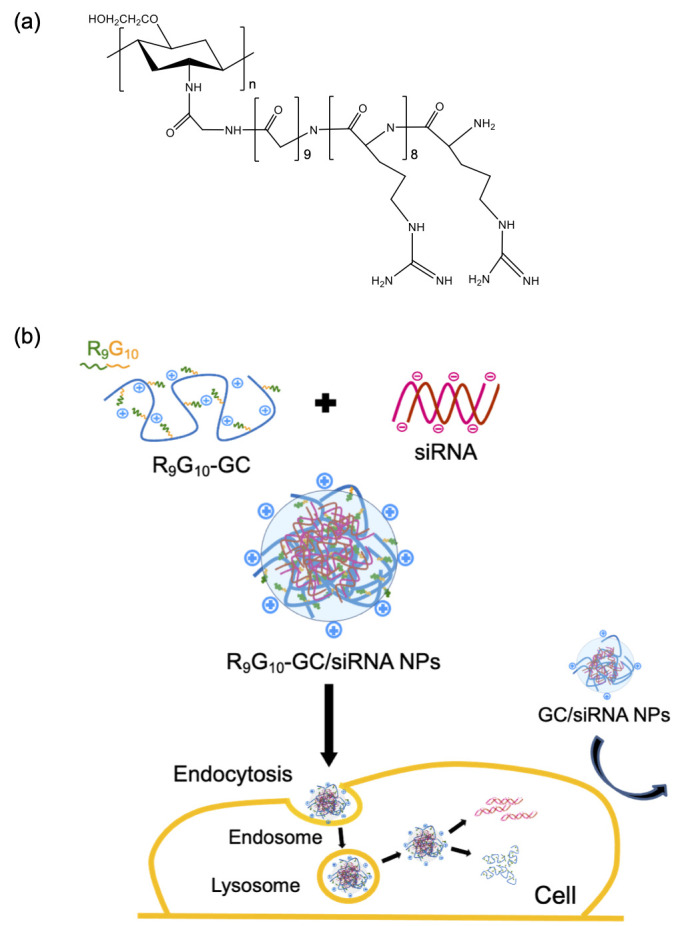
(**a**) Chemical structure of nonaarginine-modified glycol chitosan (R_9_G_10_-GC) and (**b**) schematic description for preparation of R_9_G_10_-GC/siRNA nanoparticles and their cellular uptake.

Thus, we hypothesized that the introduction of nonaarginine residues to GC could form stable complexes with negatively charged siRNA due to increased positively charged characteristics of the derivative and could enhance the gene silencing efficiency due to the trans-membrane function of the peptide. In this study, nonaarginine with 10 glycine units as a spacer arm (R_9_G_10_) was chosen and chemically introduced to the backbone of GC. A spacer arm between ligand and polymer is often required for proper interaction between the ligand and cell, due to steric hindrance caused by the macromolecular structure [40]. R_9_G_10_-GC was expected to improve the stability of GC/siRNA complexes, and improved cellular uptake and resultant transfection efficiency were also expected with the introduction of R_9_G_10_ to GC (Figure 1).

## 2. Materials and Methods

### 2.1. Materials

GC (Mw = 250 kDa, degree of deacetylation = 83%) was purchased from Sigma-Aldrich (St Louis, MO, USA), and the R_9_G_10_ peptide was supplied by Anygen (Gwangju, Korea). 2-(*N*-morpholino) ethanesulfonic acid (MES), 1-ethyl-3-(dimethylaminopropyl) carbodiimide (EDC), dimethyl sulfoxide (DMSO), potassium bromide (KBr), and 3-(4,5-dimethyl-thiazol-2-yl)-2,5-diphenyl-2H-tetrazolium bromide (MTT) were purchased from Sigma-Aldrich (St. Louis, MO, USA) and used without further purification. *N*-hydroxysulfosuccinimide (sulfo-NHS) was obtained from Pierce (Rockford, IL, USA). Dulbecco’s modified Eagle’s medium (DMEM), fetal bovine serum (FBS), phosphate-buffered saline (PBS), and penicillin-streptomycin (PS) were purchased from Gibco (Grand Island, NY, USA). Diethylpyrocarbonate (DEPC)-treated water and siRNA targeting cyclophilin B (siCypB, 5′-TGTCTTGGTGCTCTCCACC-3′) were supplied by Samchulli Pharmaceutical (Seoul, Korea) and Bioneer (Daejeon, Korea), respectively.

### 2.2. Synthesis of Peptide-Modified Glycol Chitosan

The R_9_G_10_ peptide was covalently conjugated to GC by reaction between the carboxyl group of R_9_G_10_ and amino group of GC via carbodiimide chemistry [39]. Solutions of GC (0.5 g) and R_9_G_10_ were mixed (MES buffer, pH 6.5, 100 mL), and the reaction was initiated by adding EDC (0.5 mM) and sulfo-NHS (0.25 mM). The amount of R_9_G_10_ added to the GC was varied (1.8 μmol or 14.6 μmol), and the reaction was conducted at room temperature overnight under stirring. The R_9_G_10_-GC conjugates were then purified through extensive dialysis using dialysis tubes (molecular weight cut-off, 3.5 kDa; Spectra Por, Waltham, MA, USA) against deionized water for 4 days, followed by treatment with activated charcoal for further purification, sterilization with a filter (pore size, 0.22 μm), and lyophilization.

### 2.3. Characterization of Peptide-Modified Glycol Chitosan

FT-IR spectroscopy was used to confirm the chemical conjugation between the R_9_G_10_ and GC (Nicolet IS50, Thermo Fisher Scientific; Waltham, MA, USA). The GC or R_9_G_10_-GC samples were mixed with dry KBr in a fine powder form, and a disk was prepared through compression. Each disk was scanned over a wavenumber region of 400–2000 cm^−1^ (resolution, 4 cm^−1^; scan rate, 4 mm/s). ^1^H NMR spectra were recorded using a Varian VNMRS 600 MHz spectrometer. Samples were dissolved in D_2_O ([sample] = 10 mg/mL). The degree of substitution of R_9_G_10_ conjugated to GC was calculated from elemental analysis (Thermo Finnigan Flash EA 1112, Bremen, Germany).

### 2.4. Preparation of Peptide-Modified Glycol Chitosan/siRNA Nanoparticles

R_9_G_10_-GC was dissolved in PBS (2.68 mg/mL, pH 7.4) and filtered through a 0.22-μm syringe filter. The solution was then added to an siRNA solution (50 μL) and mixed to prepare R_9_G_10_-GC/siRNA nanoparticles. siRNA was dissolved in DEPC-treated water (26.8 μg/mL) and the weight ratio of polymer to siRNA changed from 25 to 50. The nanoparticles were incubated for 30 min at room temperature before use.

### 2.5. Electrophoretic Mobility Shift Assay

Complex formations between R_9_G_10_-GC and siRNA was confirmed by an electrophoretic mobility shift assay. Nanoparticles were prepared and incubated at room temperature for 30 min before analysis. Electrophoresis was performed with a 3% agarose gel at 100 V for 30 min in tris-borate-EDTA buffer [39]. The siRNA in the gel was visualized using ethidium bromide at 365 nm. Naked siRNA was used as a control.

### 2.6. Particle Size and Zeta Potential

The mean diameter and zeta potential of the R_9_G_10_-GC/siRNA nanoparticles were measured using a dynamic light scattering method at room temperature (Nano ZS Zetasizer, Malvern Instruments, Worcestershire, UK). Nanoparticles in distilled water were loaded into a cuvette to measure their mean diameter and placed in a capillary cell to measure the zeta potential. Each experiment was performed thrice. The morphology of nanoparticles, loaded on a mica surface and purged with nitrogen, was observed by atomic force microscopy (AFM) in a non-contact mode (NX20; Park System, Suwon, Korea).

### 2.7. Cell Culture and Cytotoxicity Assay

HeLa cells were plated in 96-well tissue culture plates at a density of 5 × 10^3^ cells/well and incubated in DMEM containing 10% FBS and 1% PS at 37 °C in a 5% CO_2_ atmosphere to test the cytotoxicity of R_9_G_10_-GC/siRNA nanoparticles ([siRNA] = 50 pmol/well). MTT (10 μL) was added to each well after incubation with the nanoparticles at 37 °C for 24 h and incubated again for 4 h. After the unreduced MTT and media were removed, DMSO (100 μL) was added to each well to dissolve the formazan crystals, and the plates were incubated at room temperature for 30 min. The absorbance was measured at 540 nm using a spectrophotometer (Molecular Devices, San Jose, CA, USA). The cytotoxicity of naked siRNA, Lipofectamine™2000/siRNA nanoparticles, and GC/siRNA nanoparticles was also tested ([siRNA] = 50 pmol/well, weight ratio = 50).

### 2.8. Cellular Uptake

The cellular uptake of peptide-modified GC/siRNA nanoparticles was assessed next. The HeLa cells were seeded on a cover glass, placed in 12-well non-tissue culture plates (1 × 10^5^ cells/well), and cultured in DMEM containing 10% FBS and 1% PS at 37 °C in a 5% CO_2_ atmosphere. Fluorescein isothiocyanate (FITC)-conjugated siRNA was used to prepare nanoparticles, which were added to the plates (100 pmol/mL). The cells were fixed with 4% formaldehyde after 4 h of incubation and were treated with Vectastain^®^ containing 4′,6-diamidino-2-phenylindole (DAPI; Vector Laboratories, Burlingame, CA, USA). Lysosomal staining was performed using LysoTracker™ Red DND-99 according to the manufacturer’s instruction (50 nM; Invitrogen, Carlsbad, CA, USA). Images were captured using a fluorescence microscope (Nikon Instruments, Melville, NY, USA).

### 2.9. Gene Silencing

The in vitro gene silencing efficacy of R_9_G_10_-GC/siRNA nanoparticles was assessed in the HeLa cells. Cells were plated in 12-well tissue culture plates (1 × 10^5^ cells/well) and incubated in DMEM containing 10% FBS and 1% PS at 37 °C in a 5% CO_2_ atmosphere to assess the gene silencing efficiency of R_9_G_10_-GC/siRNA nanoparticles. The medium was replaced with DMEM without FBS on the day of transfection. Cells were treated with nanoparticles (100 μL) containing siRNA (200 pmol) and incubated for 4 h. The medium was then replaced with DMEM containing 10% FBS, and the cells were incubated at 37 °C in a 5% CO_2_ atmosphere for 44 h. Quantitative gene expression of cyclophilin B (CypB) was evaluated using real-time SYBR Green PCR technology (ABI PRISM 7500 Real-Time PCR System, Applied Biosystems, Foster City, CA, USA). RNA was isolated from the HeLa cells using the RNAiso kit (Takara, Tokyo, Japan). The gene expression level was determined by comparison with that of the reference gene (GAPDH). The sequences of the primers used were as follows: CypB (160 bp), 5′-TGGAGAGCACCAAGACAGACA-3′ and 5′-GTCGACAATGATGACATCCTTCA -3′; GAPDH (86 bp), 5′-GGCAAATTCAACGGCACAGT-3′ and 5′-GGGTCTCGCTCCTGGAAGAT-3′.

### 2.10. Statistical Analysis

All data are presented as mean ± standard deviation. Statistical analyses were performed using the Student’s *t*-test. ** *p*-values < 0.01 and *** *p*-values < 0.001 were regarded as statistically significant.

## 3. Results and Discussion

### 3.1. Characterization of Peptide-Modified Glycol Chitosan

Peptide-modified glycol chitosan (R_9_G_10_-GC) was synthesized by coupling the carboxyl group of R_9_G_10_ with the amino group of GC via carbodiimide chemistry. EDC is a representative zero-length cross-linker and its conjugation reaction can be improved in the presence of sulfo-NHS because of the prolonged stability of the active ester intermediate against hydrolysis in aqueous solution when compared with the EDC [41]. Unreacted peptide and EDC were removed from the reaction solution by extensive dialysis.

**Figure 2:**
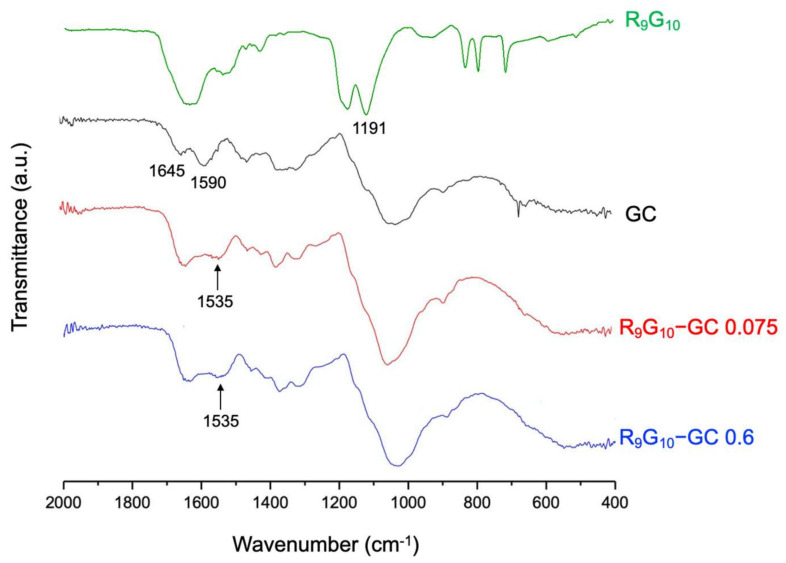
Fourier transform infrared spectroscopy (FT-IR) spectra of R_9_G_10_-GCs with different substitution degrees. The new amide bonds in R_9_G_10_-GCs after conjugation reaction were observed at 1535 cm^−1^ and indicated by arrows.

**Figure 3:**
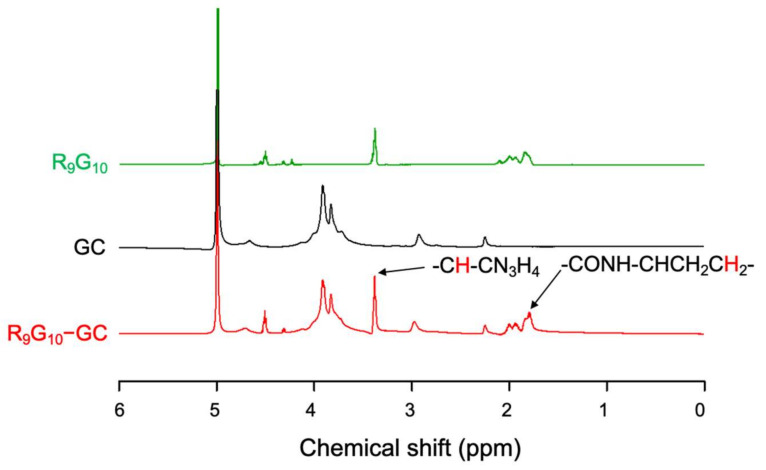
Nuclear magnetic resonance (^1^H NMR) spectra of R_9_G_10_, GC, and R_9_G_10_-GC 0.6.

The conjugation of R_9_G_10_ and GC was identified by FT-IR and ^1^H NMR spectroscopy. The bands of C = O stretching at 1645 cm^−1^ (amide I) and NH bending at 1590 cm^−1^ (primary amine) were observed for the GC by FT-IR spectroscopy [42]. A new peak corresponding to the amide bond was observed at 1535 cm^−1^ for R_9_G_10_-GCs (Figure 2), indicating the successful covalent linkage between R_9_G_10_ and GC. In addition, the band of C(O)–O stretching of R_9_G_10_ at 1191 cm^−1^ disappeared after conjugation with GC [36,43]. The new peaks were also observed by ^1^H NMR spectroscopy at 1.8 ppm (–CONH–CHCH_2_CH_2_–) and at 3.3 ppm (–CH–CN_3_H_4_) for R_9_G_10_-GC (Figure 3) [36,44]. The degree of substitution (DS), which indicates the molar ratio of R_9_G_10_ per 100 glucosamine residues in GC, was confirmed through elemental analysis (Table 1). The conjugation efficiency, calculated from the theoretical and actual DS values, was >90% for the R_9_G_10_-GCs used in this study.

### 3.2. Interactions between siRNA and Peptide-Modified Glycol Chitosan

**Figure 4:**
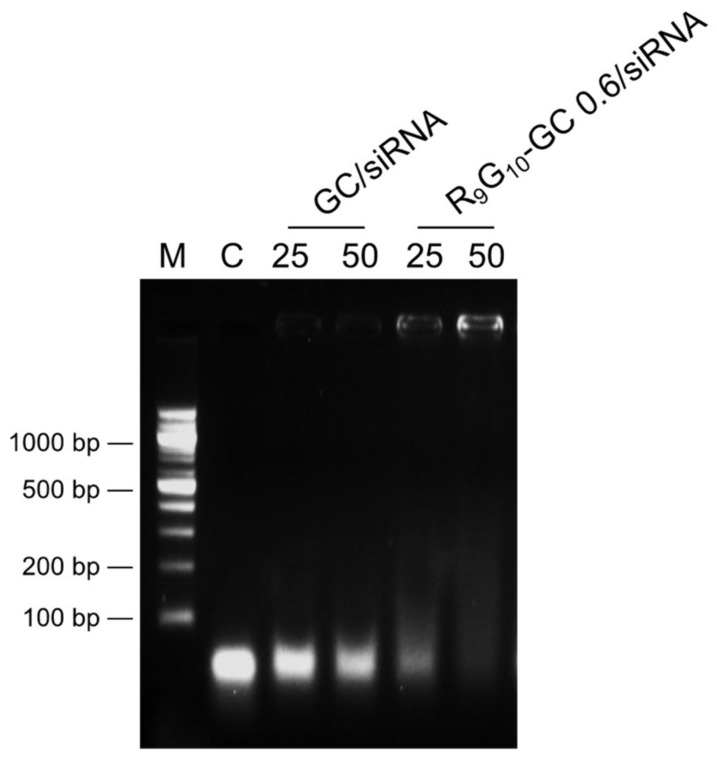
Gel retardation assay of R_9_G_10_-GC/siRNA nanoparticles (M, DNA markers; C, naked siRNA). Lanes marked with 25 and 50 indicate R_9_G_10_-GC/siRNA ratios (weight ratio). GC/siRNA nanoparticles were also prepared.

Gel electrophoresis was performed to confirm the formation of the R_9_G_10_-GC/siRNA complexes. R_9_G_10_-GC/siRNA nanoparticles were formed at weight ratios of 25 and 50. The weight ratio of GC to siRNA approximately equals half the value of an N/P ratio. The movement of siRNA was not remarkably retarded when GC was used to form complexes. However, R_9_G_10_-GC 0.6 was useful to retard the movement of siRNA in the gel, and the weight ratio of 50 was much more effective in forming complexes than that with a weight ratio of 25 (Figure 4). The smearing bands observed in Figure 3 may result from incomplete binding between polymer and siRNA. R_9_G_10_-GC 0.075 was not effective for complex formation when mixed with siRNA (data not shown). This finding suggests that the increased positive charge of R_9_G_10_-GC with higher DS could be critical for complex formation with negatively charged siRNA. The ability of cationic polymers to interact with anionic siRNA could be importantly related to the size, morphology, cellular uptake, and gene silencing efficiency of the nanoparticles.

### 3.3. Size and Surface Charge of Peptide-Modified Glycol Chitosan/siRNA Nanoparticles

**Figure 5:**
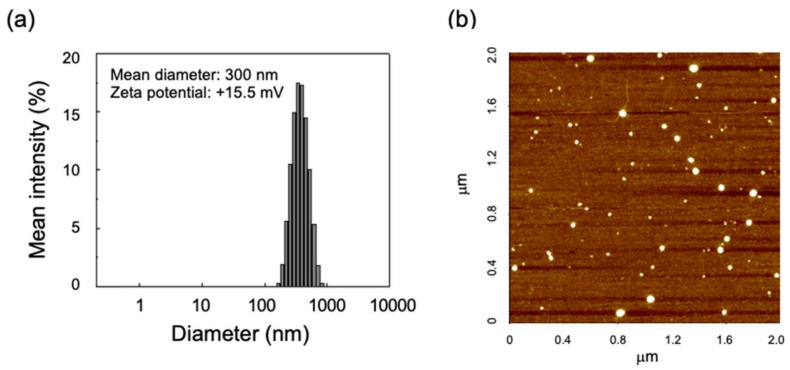
(**a**) Size distribution of R_9_G_10_-GC 0.6/siRNA nanoparticles measured by the dynamic light scattering method at 25 °C and (**b**) atomic force microscopy (AFM) image of the nanoparticles (R_9_G_10_-GC 0.6/siRNA = 50, weight ratio).

The size (i.e., hydrodynamic diameter) and surface charge of the nanoparticles were investigated (Figure 5a). The mean diameter of the R_9_G_10_-GC 0.6/siRNA nanoparticles was 300 nm (PDI = 0.232). The representative image of nanoparticles was obtained by AFM, suggesting round shape of the R_9_G_10_-GC 0.6/siRNA nanoparticles (Figure 5b). The zeta potential of R_9_G_10_-GC 0.6/siRNA nanoparticles was +15.5 mV, which was very close to that of chitosan/siRNA nanoparticles (+15.8 mV) as previously reported [45]. The zeta-potential of oligoarginine-modified chitosan/siRNA nanoparticles could be controlled by different conjugation degrees [39]. However, we were not able to observe changes in the zeta potentials due to the very limited range of conjugation degrees in this study. The GC only or R_9_G_10_-GC 0.075 did not form stable nanoparticles, and the size and zeta potential values could not be determined. This finding may indicate that the zeta potential could be substantially improved using R_9_G_10_-GC when compared with GC. The size and surface charge of gene carriers are key factors for cellular uptake and resultant transfection in cells. Furthermore, it was demonstrated that the positively charged surface of nanoparticles facilitated their adhesion to cell membranes and improved their potential as drug delivery carriers [46,47]. Based on the aforementioned results, R_9_G_10_-GC 0.6/siRNA nanoparticles with a weight ratio of 50 were used for cytotoxicity and gene silencing analyses.

### 3.4. Cytotoxicity

**Figure 6:**
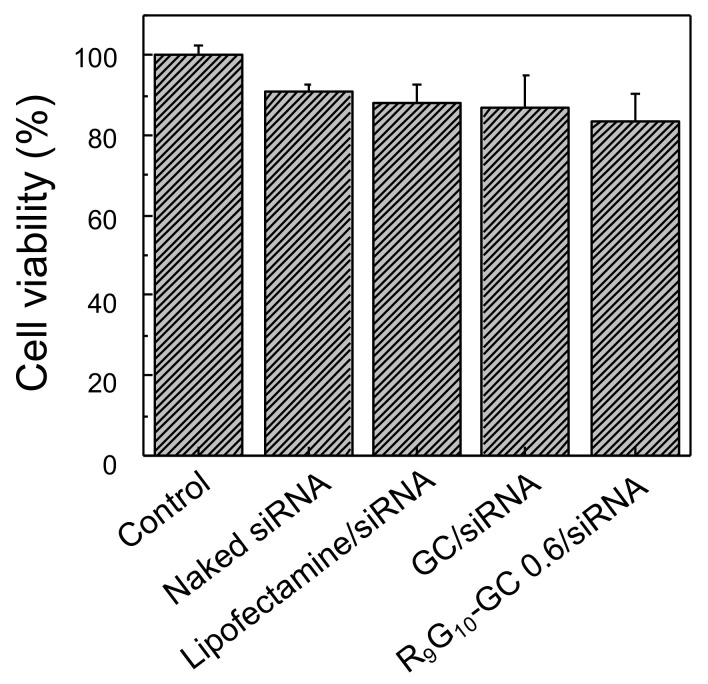
Cytotoxicity of various nanoparticles was evaluated with HeLa cells by the MTT assay. The value was normalized to that of the untreated cells used as a control ([siRNA] = 50 pmol/well, weight ratio = 50, *n* = 6).

The viability of the HeLa cells treated with R_9_G_10_-GC 0.6/siRNA nanoparticles was quantitatively assessed using the MTT assay. The value was normalized to that of the untreated cells used as a control. R_9_G_10_-GC 0.6/siRNA nanoparticles showed no significant decrease in cell viability (83.7 ± 6.5%) when compared with naked siRNA (90.8 ± 2.2%), Lipofectamine/siRNA nanoparticles (88.3 ± 4.2%), and GC/siRNA nanoparticles (86.9 ± 7.9%) (Figure 6). This may be attributed to the inherent low toxicity of GC and low DS of R_9_G_10_ in R_9_G_10_-GC.

### 3.5. Cellular Uptake

**Figure 7:**
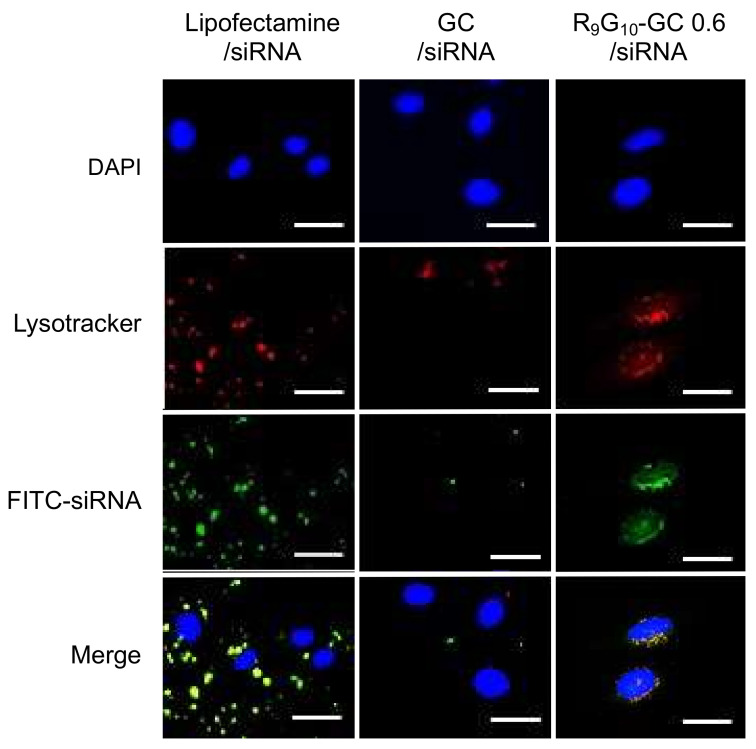
Confocal microscopic images of HeLa cells treated with various types of nanoparticles (scale bar, 50 μm). Fluorescein isothiocyanate (FITC)-siRNA was used to prepare nanoparticles (green) and Lysotracker was used to stain lysosome in the cells (red).

The uptake of nanoparticles prepared with nonaarginine-modified GC and FITC-labeled siRNA in the HeLa cells was investigated. Internalization of FITC-siRNA (green) in the lysosome (red) was observed for Lipofectamine/siRNA and R_9_G_10_-GC/siRNA nanoparticles (Figure 7). The cellular uptake of R_9_G_10_-GC/siRNA nanoparticles was also higher than that of GC/siRNA nanoparticles. This finding may be because the nonaarginine peptide plays a critical role as a CPP when delivering nanoparticles into cells. Naked siRNA alone is not substantially taken up by cells due to the negatively charged cell membrane. It has been reported that the trans-membrane function of the nonaarginine peptide significantly can enhance the cellular uptake and resultant transfection efficiency [39,45].

### 3.6. Gene Silencing

**Figure 8:**
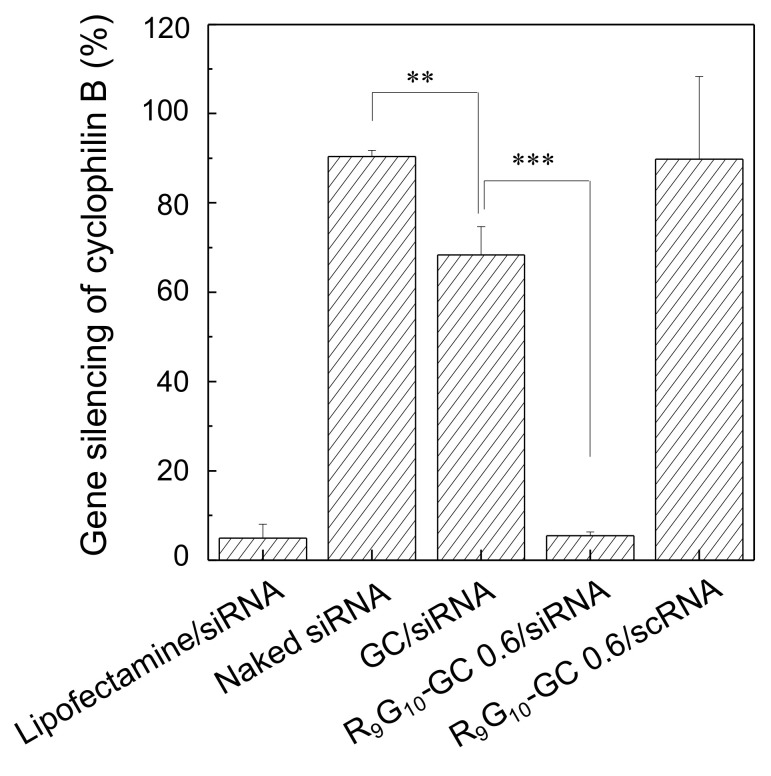
Quantitative gene silencing efficiency of R_9_G_10_-GC/siCypB nanoparticles, which was normalized to GAPDH expression. Naked siCypB and scrambled siRNA (scRNA) were also tested (*n* = 5, ** *p* < 0.01, *** *p* < 0.001).

The gene silencing efficiency of nanoparticles containing siCypB was quantitatively analyzed by real-time PCR (Figure 8). The gene silencing efficiency of R_9_G_10_-GC 0.6/siRNA nanoparticles was substantially higher than that of GC/siRNA nanoparticles. The gene silencing efficiency of R_9_G_10_-GC 0.6/siRNA was comparable to that of Lipofectamine, a commercially available liposome used as a positive control (approximately 95%). This finding may be explained by the role of the nonaarginine peptide as a cell-penetrating peptide. Gene silencing was negligible for naked siRNA and R_9_G_10_-GC 0.6/scRNA complexes.

Introduction of oligoarginine to chitosan was useful to enhance the transfection efficiency of chitosan/siRNA nanoparticles because of the CPP effect of the nanoparticles [39,45]. However, oligoarginine-modified chitosan is still soluble under acidic conditions. Although GC is known to be highly soluble under physiological conditions, GC alone lacks the capability to effectively form complexes with siRNA. Thus, conjugation of oligoarginine to GC enables nanoparticle preparation with siRNA under physiological conditions, which may be advantageous to be exploited as a gene delivery vehicle. The enhanced transfection efficiency of R_9_G_10_-GC 0.6/siRNA could also be attributed to protection of the siRNA from degradation using R_9_G_10_-GC similar to the use of cationic polymers [39,48].

## 4. Conclusions

We demonstrated that nonaarginine-modified glycol chitosan was useful for siRNA delivery in vitro. R_9_G_10_-GC was successfully synthesized by introducing R_9_G_10_ to GC via carbodiimide chemistry. The conjugate formed stable electrostatic complexes with negatively charged siRNA. The DS of R_9_G_10_ in R_9_G_10_-GC as well as the amount of R_9_G_10_-GC in R_9_G_10_-GC/siRNA nanoparticles were key factors to regulate the characteristics of the nanoparticles. The in vitro cellular uptake and gene silencing efficiency of R_9_G_10_-GC 0.6/siRNA nanoparticles were substantially enhanced compared with GC/siRNA nanoparticles while maintaining a low level of cytotoxicity. This finding can be attributed to the increased positively charged characteristics of the derivative and the trans-membrane function of the peptide. This approach may provide a useful means for the development of novel polysaccharide-based delivery carriers for gene therapy applications.

## Figures and Tables

**Table 1:** Characteristics of nonaarginine-modified glycol chitosan samples.

Sample	Theoretical DS ^a^	Actual DS	Conjugation Efficiency (%)
R_9_G_10_-GC 0.075	0.075	0.071	94.6
R_9_G_10_-GC 0.6	0.6	0.544	90.7

^a^ DS indicates the degree of substitution, which was defined as the molar ratio between R_9_G_10_ and 100 glucosamine residues of GC. The number behind a sample name indicates the theoretical DS value.

## Data Availability

The data presented in this study are available upon request from the corresponding author.
